# PBK correlates with prognosis, immune escape and drug response in LUAD

**DOI:** 10.1038/s41598-023-47781-7

**Published:** 2023-11-22

**Authors:** Hongyu Ma, Jing Zhang, Yan Shi, Ziqiang Wang, Wenhu Nie, Jingjing Cai, Yinglong Huang, Bin Liu, Xiaojing Wang, Chaoqun Lian

**Affiliations:** 1 Department of Clinical Medicine, Bengbu Medical College, Bengbu, 233000 China; 2 Department of Biochemistry and Molecular Biology, School of Laboratory Medicine, Bengbu Medical College, Bengbu, 233000 China; 3 Department of Genetics, School of Life Sciences, Bengbu Medical College, Bengbu, 233000 China; 4 Department of Pulmonary and Critical Care Medicine, Anhui Province Key Laboratory of Clinical and Preclinical Research in Respiratory Disease, Molecular Diagnosis Center, First Affiliated Hospital of Bengbu Medical College, Bengbu, 233000 China; 5 Research Center of Clinical Laboratory Science, Bengbu Medical College, Bengbu, 233000 China; 6 Department of Respiratory and Critical Care Medicine, Fuyang People’s Hospital, Fuyang, 236000 China

**Keywords:** Cancer, Computational biology and bioinformatics, Immunology, Oncology

## Abstract

PBK (PDZ-binding kinase) is a protein-coding gene that encodes a serine/threonine protein kinase associated with the dual-specific mitogen-activated protein kinase (MAPKK) family. Overexpression of this gene is closely linked to tumor development. In this study, we aimed to investigate the role of PBK in lung adenocarcinoma (LUAD) progression, prognosis, and immune evasion. We conducted a pan-cancer analysis of PBK to examine its expression and prognostic value. In the LUAD cohort, we analyzed PBK expression, prognosis, mutational features, and immune infiltration in groups with different PBK expression levels. We constructed a PBK-associated genomic model, integrated it into a nomogram, and compared high and low-risk subgroups. In our pan-cancer analysis, PBK was significantly upregulated, particularly in LUAD patients, and displayed poor prognosis. The high PBK expression group had many deletion mutations but still showed gene upregulation. Immune infiltration analysis indicated that PBK-triggered immune escape in the high expression group might relate to antigen presentation, dendritic cell, and CD8+ T cell infiltration. We constructed a 5-gene prognostic model and a nomogram to quantify individual survival probabilities. The PBK-associated gene prognostic model reliably predicted patient prognosis and drug response. Our findings offer new insights into PBK-induced immune escape and targeted therapy during LUAD development, providing valuable suggestions for clinical treatment approaches.

## Introduction

Non-small cell lung cancer (NSCLC) is one of the most common cancers worldwide, accounting for approximately 55–60% of lung cancer deaths^1^. Lung adenocarcinoma (LUAD) has now become the most prevalent subtype of NSCLC and a significant cause of cancer-related mortalities globally. Currently, even patients with good clinical stage lung adenocarcinoma have a poor prognosis, showing an overall survival rate of about 70% at 5 years post-surgical resection^2^. This indicates that there is a need for further optimization of lung adenocarcinoma treatments, including adjuvant drug-targeted therapy and immunotherapy. However, patient-specific optimized treatment options are still limited. While chemotherapy remains a recommended clinical treatment, only patients with more discernible mutations are eligible for targeted therapy. For instance, oncogenic driver mutations in genes encoding anaplastic lymphoma kinase (ALK) or epidermal growth factor receptor (EGFR) can render lung tumors sensitive to targeted tyrosine kinase inhibition^3^. Thus, there is an urgent requirement for screening new molecular targets of LUAD to optimize patient selection.

There is growing evidence that the tumor microenvironment (TME) plays a crucial role in promoting lung cancer development and progression^4^. In one study, the spatial distribution of CD8+ T cells was observed across three immunophenotypic gradients: immune inflammation (II TIME), immune desert (ID TIME), and immune rejection (IE TIME)^5,6^. Inflammatory tumors exhibit intact IFNg signaling, PD-L1 expression, TILs, B cells, antigen expression, intact HLA, and tumor cell surface MHC class I expression, leading to a large inflammatory cell infiltrate in the tumor and generally resulting in a better response to immune checkpoint inhibitor (ICI) therapy^7^. However, patients with immune rejection tumors or immune deserts often exhibit poor responses to ICI therapy due to lack of T-cell infiltration and low MHC class I expression. The connection between different immune subtypes of tumors and targeted drug therapy is significant, leading to the development of individualized treatment strategies.

The PBK gene encodes a serine/threonine protein kinase associated with the bispecific mitogen-activated protein kinase (MAPKK) family^8,9^. It is involved in lymphocyte activation and is active only during mitosis. Phosphorylation of PBK can form a complex with TP53, causing TP53 instability and reducing G2/M checkpoints during doxorubicin-induced DNA damage^10^. Some evidence indicates that PBK expression levels correlate with hypoxia in various tumors and affect processes such as tumorigenicity and progression^11^. Meanwhile, tumor immune escape in patients contributes to malignant proliferation and metastasis without killing cancer cells, often making immunotherapy and chemotherapy ineffective or resistant. PBK interaction with HIF1 can upregulate programmed death ligand 1 (PD-L1) and vascular endothelial growth factor (VEGFA) expression via the hypoxia-inducible factor 1α (HIF-1α) pathway^12–14^. Consequently, hypoxia-mediated tumor immune escape is likely to occur. In general, PBK can influence the immune phenotype and immune microenvironment of tumor patients, but the exact effects of PBK on tumor immune status, microenvironment, and patient response to immunotherapy require further investigation.

## Materials and methods

### Pan-cancer data source and processing

The TCGA database (https://portal.gdc.cancer.gov/) stores current RNA-seq data for 33 cancer types for download by investigators, and we also obtained prognostic information for the corresponding cancers. RNA sequencing data values from the TCGA dataset were uniformly converted to transformed log2(TPM + 1) values. Data from patients with partial somatic mutations and copy number alterations were downloaded from the cbioportal.org online tool (https://www.cbioportal.org) and GDC for studying the relationship between CNA and DNA alterations in patients with and without disease (CNA and mutations). We also obtained pan-cancer TMB data for mapping the correlation between PBK and TMB, and the correlation analysis was performed using the Spearman method. In combination with the obtained sample mutation data we also downloaded from GDC a level 4 simple nucleotide variation dataset of all TCGA samples processed by MuTect2 software to demonstrate the landscape of PBK gene alterations.

### Prognostic analysis

At the prognostic level, the prognostic impact of PBK gene in pan-cancer was analyzed by coxph function to analyze the relationship between gene expression and prognosis in each tumor. And the association between PBK gene expression and overall survival (OS), disease-specific survival (DSS), disease-free survival (DFI) and progression-free survival (PFI) was objectively assessed, and the significance of PBK expression and prognosis was obtained by Logrank test. Multivariate Cox regression and Kaplan–Meier analyses to construct PBK-associated gene models were performed by the R packages "survminer" and "survivor".

### Sources of validated data related to the PBK gene

The GSE116959^15^, GSE19188^16^, CPTAC-LUAD (https://cptac-data-portal.georgetown.edu/cptac) datasets were used to validate PBK transcript and protein level expression, and PBK-associated gene prognostic modeling was validated by GSE37745^17^, and GSE72094^18^. The GSE41271^19^ dataset was used to validate immune infiltration between high and low PBK groups. HPA database(https://www.proteinatlas.org/) for obtaining immunohistochemical staining data of PBK proteins in tissue sections from LUAD patients. The DepMap database(https://depmap.org/portal/) provides experimental data of genes in a large number of cancer cell lines after CRISPR/RNAi, reflecting the effects of genes on the survival and proliferation of specific cell lines^20^. Here we obtained the effect data and expression data of PBK after RNAi in each LUAD cell line through Depmap.

The corresponding probe IDs were mapped to gene symbols one by one using R software according to the annotation file of the corresponding platform for each dataset, and expression measurements for multiple or all probes corresponding to the same gene were mean-combined to obtain expression values for individual genes.

### Enrichment analysis

Hallmark gene sets and GSEA reference gene sets were obtained from the MSigDB database on the GSEA website (http://www.gsea-msigdb.org/gsea/index.jsp), Version including Reactome, PID and Wikipathways database "MSigDB v2022.1.Hs". GSEA analysis was used to investigate differences in activated signaling pathways between high and low PBK samples. Gene ontology (GO) and Kyoto Encyclopedia of Genes and Genomes (KEGG) enrichment analysis of differential genes between PBK high and low expression groups was performed using the clusterProfiler package, and the gene set "c2.cp.kegg.v7.4.symbols.gmt" and “c5.go.v2023.2.Hs.symbols.gmt” was as a reference gene set. Changes in pathway activity of Hallmark, Mariathasan and partial immune infiltration-associated gene sets were assessed by the ssGSEA algorithm in the GSVA package^21^.

### Assessment of the immunological profile between high and low PBK gene expression

Twenty-eight representative immune cells curated by Tumor-Immune System Interaction (TISIDB) were used to explore the level of immune infiltration^22^. The ESTIMATE algorithm was used to confirm the overall infiltration of stromal and immune cells in LUAD tissues and return the corresponding infiltration scores. Next, we used the xCELL, TIMER, CIBERSORT, MCPCounter, EPIC, and quanTIseq algorithms^23–29^ to calculate the level of stromal and immune cell infiltration in LUAD patients. Immunomodulation-related genes (chemokine-receptors, MHC, immunostimulatory molecules, etc.) and immune checkpoint genes were obtained from previous studies by Charoentong et al., Auslander et al.^30,31^, from which immune-related signature genes were also confirmed and compared with those in TISIDB. The analysis of PBK gene correlation with the 7-step cycle process of cancer immunity was based on that obtained in the TIP (http://biocc.hrbmu.edu.cn/TIP/)^32^. The evaluation of processes such as angiogenesis and EMT was obtained from the genome proposed by Mariathasan et al.^33^.

### DEGs between high and low PBK expression groups

LUAD patients were divided into high PBK-expressing and low PBK-expressing subgroups according to median PBK expression. Genes differentially expressed between subgroups were identified using the Deseq2 package. We used the |logFC|> 1.5 and adjusted P value < 0.05 conditions as criteria for screening differential gene thresholds between PBK differentially expressed groups.

### Identification of associated genes from PBK differential genes for genomic modeling and histogram construction

Differential genes between different expression groups of PBK obtained from the TCGA cohort were further screened by univariate Cox regression models for differential genes between PBK expression groups associated with LUAD prognosis. Screening was performed according to a threshold of P value < 0.05, and correlations between PBK and differential genes were analyzed by importing PBK and differential genes through the String database, with an intermediate confidence level set at 0.4. Differential prognostic genes associated with PBK that met the requirements were identified based on an intermediate confidence level > 0.4. The differential prognostic genes associated with PBK were subsequently used to construct a prognostic model for PBK-associated differential genes by multivariate Cox regression, and the PBK-associated genomic model was calculated as follows. Risk score = EXP(Gene1) * coefficient(1)  + … + EXP(GeneN) * coefficient(N), firstly EXP(GeneN) denotes the expression of the characteristic gene, where N denotes the number of PBK-related characteristic genes, and finally coefficient denotes the gene regression coefficient in multivariate Cox regression analysis. Afterwards, the risk score of each LUAD patient was calculated based on this formula. LUAD patients were further divided into high-risk and low-risk groups by the median of the patient's risk score in all subsequent prognostic model analyses. Kaplan–Meier curves to demonstrate the differences in prognosis between the two groups Prognostic heat map demonstrates the patients' risk scores, survival information and gene expression. Independent predictive relationships between risk scores of PBK-related gene models reflecting LUAD patients and age, sex, stage, T, N and M clinical characteristics and overall survival were performed by multivariate Cox. ROC curves were plotted using the survROC software package, and ROC curves were used to plot and assess the prognostic value of survival and Nomogram at 1, 3 and 5 years. Finally we plotted calibration curves for the Nomogram to determine the agreement between the actual survival probabilities and the survival probabilities predicted by the Nomogram.

### Drug sensitivity analysis

Preliminary drug prediction was performed using 60 different cancer cell lines from 9 different tumors from the NCI-60 CellMiner database (https://discover.nci.nih.gov/cellminer)^34^. The relationship between PBK and its associated genetic models and drug sensitivity was investigated using the Spearman method. oncoPredict was used to further validate the sensitivity to chemotherapeutic drugs in the risk group of LUAD patients in NCI-60. oncoPredict can be continuously trained based on a ridge regression model using expression data from cancer cell lines in the database and drug response data. Sensitivity scores are then generated, and the scores obtained are used to predict the half-maximal inhibitory concentration (IC50) of all drugs in LUAD patients^35^.

### Statistical analysis

The Kruskal–Wallis test was used in the pan-cancer analysis to compare gene expression in different tissues and cancerous tissues, and the Wilcoxon test was used to compare differences between risk groups for high and low PBK expression and PBK-associated gene models. Univariate and multivariate Cox regression analyses were used to assess the independent prognostic value of PBK and PBK-associated gene models for survival. log-rank test was used for Kaplan–Meier curves and clinical characteristics. Significance P < 0.05 was considered statistically significant. All R packages mentioned above were run under R software version v4.2.1.

### Ethics approval and consent to participate

All data are from public databases and an ethics approval is waived.

## Result

### PBK expression and prognosis in pan-cancer

**Figure 1:**
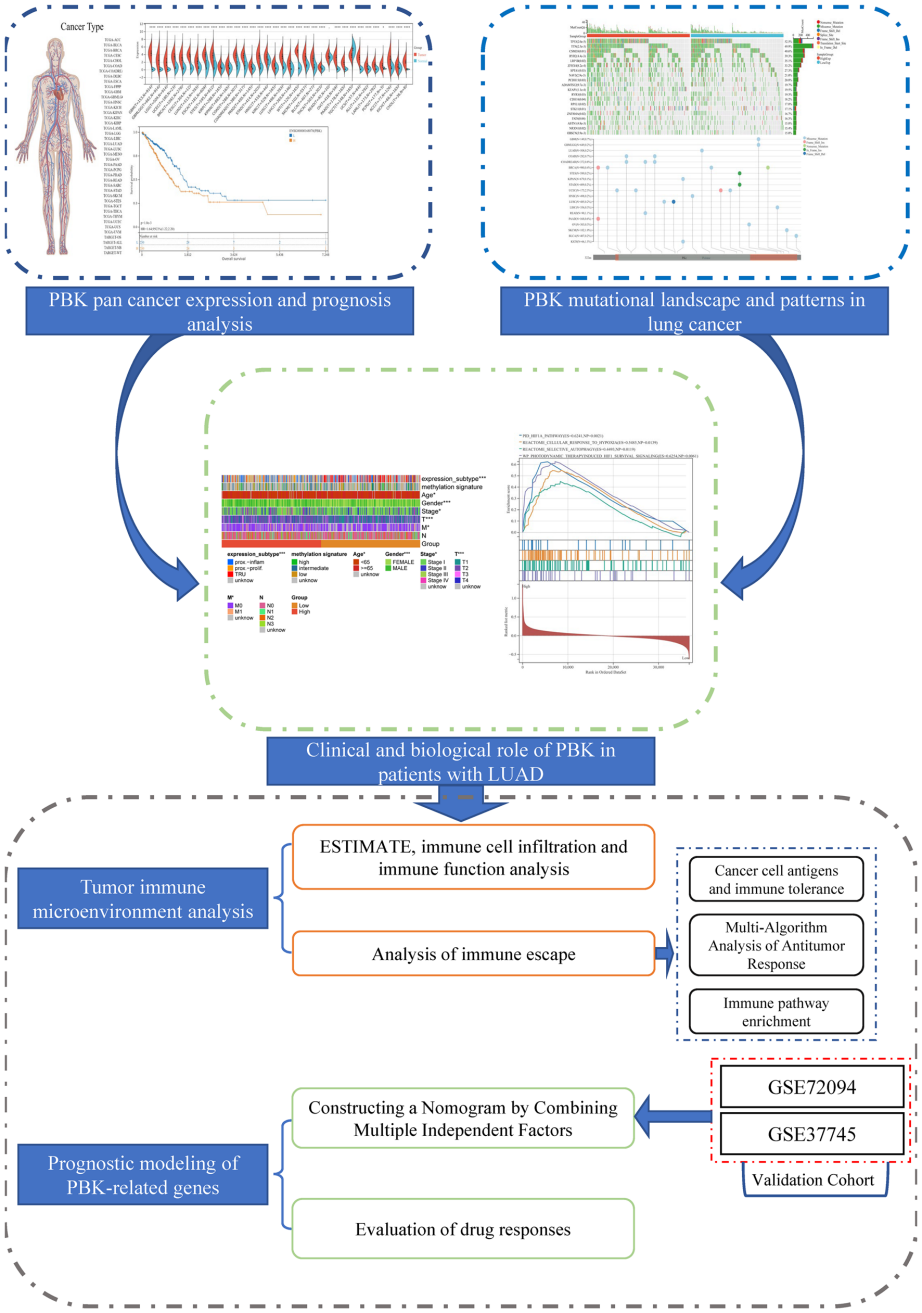
The basic workflow of this study. Image generated in Microsoft PowerPoint (version 16.0), “https://www.microsoft.com/powerpoint”.

**Figure 2:**
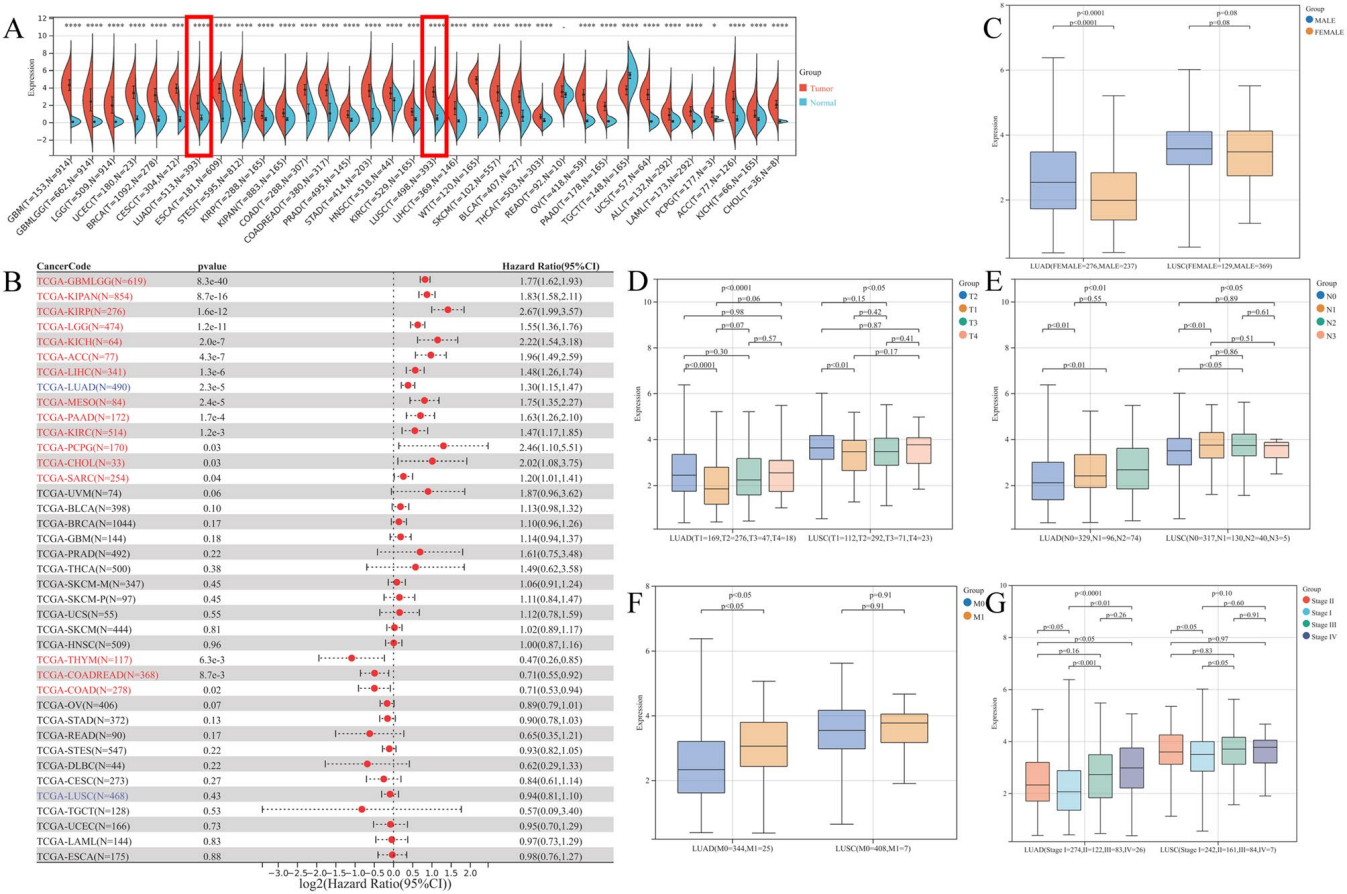
Expression and prognostic analysis of PBK in pan-cancer. (**A**) Cross-sectional plots showing the difference in PBK expression in 34 tumors versus the corresponding normal tissues. (**B**) Forest plot showing the relationship between PBK gene expression and overall survival (OS) prognosis in each tumor, with prognostic significance obtained by Logrank test. (**C–G**) Differential analysis of PBK expression in clinical characteristics (gender, T, N, M and stage) of lung adenocarcinoma patients.

Our basic workflow for this study is shown in the figure (Fig. 1). The downloaded pan-cancer dataset was normalized and merged, and 26 tumor types with PBK gene expression in each sample were further extracted from the dataset. The remaining tumor types were excluded due to the small number of samples in which significance could not be observed. We observed a significant upregulation in lung tumors (Fig. 2A). In the prognostic section, we first obtained the significance of the prognostic analysis by analyzing the prognostic relationship between PBK gene expression and overall survival OS in 26 cancer types by Logrank test, and finally observed significant upregulation in 13 tumor types (TCGA-GBMLGG (N = 655, p = 1.2e−39, HR = 1.75 (1.60, 1.91)), TCGA-LGG (N = 503, p = 4.2e−11, HR = 1.52 (1.34, 1.73)), TCGA-LUAD (N = 500, p = 7.2e−5, HR = 1.28 (1.13, 1.44)), TCGA-SARC (N = 258, p = 0.03, HR = 1.20 (1.02, 1.41)), TCGA-KIRP (N = 285,p = 1.6e−12, HR = 2.67 (1.99, 3.57)), TCGA-KIPAN (N = 878, p = 1.0e−15, HR = 1.82 (1.58, 2.10)), TCGA-KIRC (N = 528, p = 1.1e−3, HR = 1.47 (1.17, 1.84)), TCGA-LIHC (N = 362, p = 2.6e−6, HR = 1.45 (1.24, 1.70)), TCGA-MESO (N = 85, p = 8.2e−6, HR = 1.80 (1.39, 2.33)), TCGA-PAAD (N = 177, p = 1.3e−4, HR = 1.63 (1.27, 2.10)), TCGA-PCPG (N = 177, p = 0.03, HR = 2.46 (1.10, 5.51)), TCGA-ACC (N = 77, p = 4.3e−7, HR = 1.96 (1.49, 2.59)), TCGA-KICH (N = 65, p = 2.0e−7, HR = 2.22 (1.54, 3.18))) with poor prognosis of high expression in three tumor types (TCGA-COAD (N = 282, p = 0.02, HR = 0.71 (0.53, 0.94)), TCGA-COADREAD (N = 373, p = 8.7e−3, HR = 0.71 (0.55, 0.92)), TCGA-THYM (N = 118, p = 6.3e−3, HR = 0.47 (0.26, 0.85))) with poor prognosis in low expression, PBK in lung tumors is shown in blue in Fig. 2B. Secondly, we also analyzed PBK expression in different clinical characteristics (sex, T, N, M and stage) (Fig. 2C–G), further complemented by the analysis of the impact of PBK on lung tumor prognosis in disease-specific survival, disease-free survival, progression-free survival and survival curves found that PBK gene was more relevant in lung adenocarcinoma patients (Supplementary Fig. 1A–G). Consistently, we validated PBK expression in the GSE116959, GSE19188 and CPTAC-LUAD cohort with increased PBK mRNA and protein levels in tumor samples (Supplementary Fig. 1H–J).

The results were consistent in that in vitro experimental data obtained from the HPA and Depmap databases were able to be seen. Immunohistochemical staining of tissue sections showed higher levels of PBK protein expression than in normal tissues(Supplementary Fig. 2A–D), while in LUAD-associated cell lines, we observed a strong correlation between cell line effects brought about by the PBK gene and PBK gene expression after subjecting the PBK gene to RNAi, especially in metastatic types of cell lines(Supplementary Fig. 2E, F).

### Mutational landscape of PBK gene in LUAD

**Figure 3:**
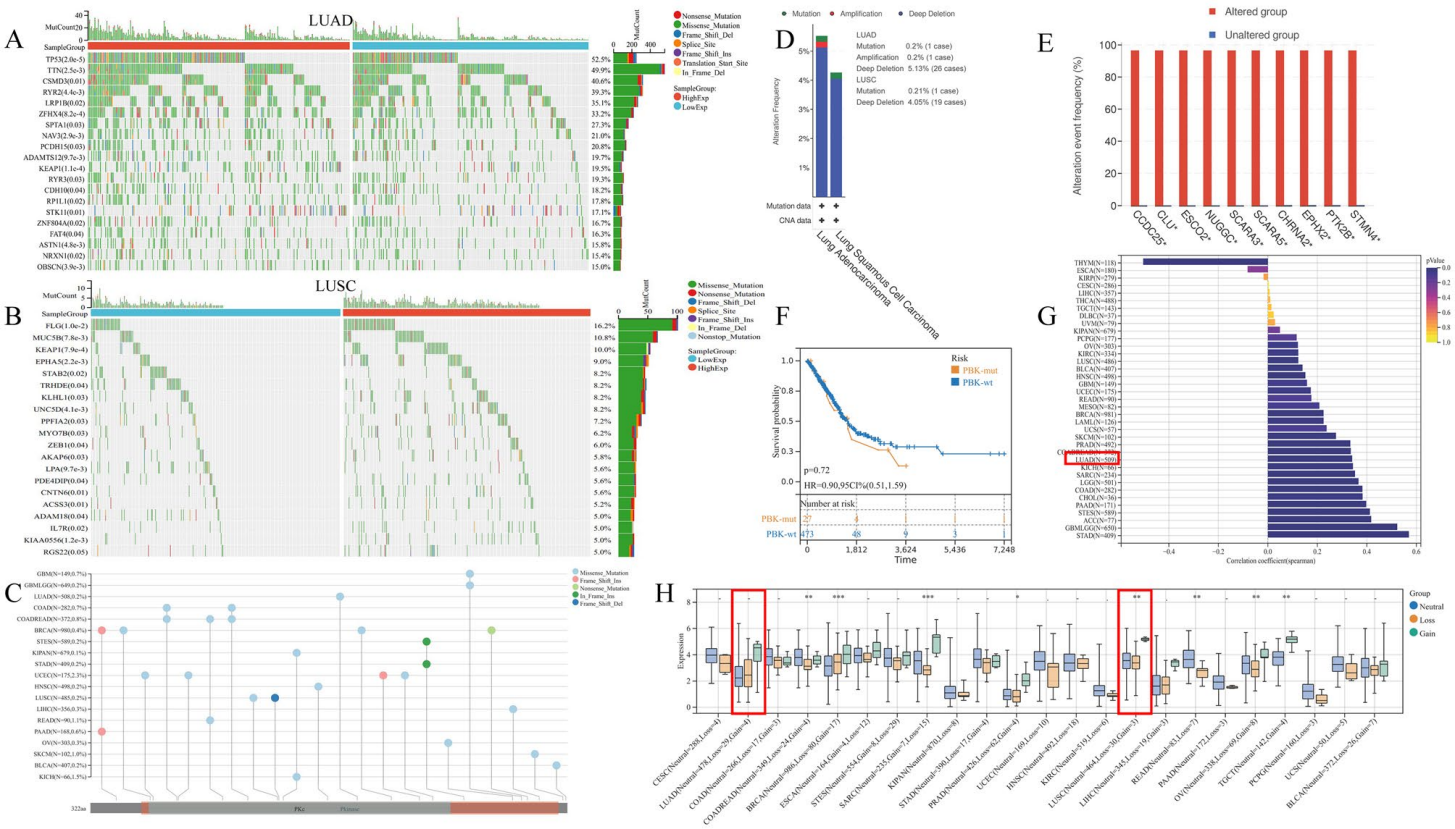
Genetic alterations and effects of PBK in lung cancer. (**A,B**) Waterfall plots showing the difference in mutation frequency in mutated samples in LUAD and LUSC. (**C**) Bar graph showing the integration of mutational information and PBK protein structural domain information in pan-cancer. (**D**) Frequency of genetic alterations in lung cancer patients in TCGA. (**E**) Histogram of the proportion of genetic changes between altered and unaltered pattern groups in lung cancer. (**F**) Comparative survival analysis between PBK mutations and PBK-wt. (**G**) Pan-cancer analysis of correlation between PBK expression and tumor mutational load, correlation analysis using Spearman method. (**H**) Differential expression of PBK in different mutation types in pan-cancer CNV.

For our preliminary analysis of the genomic signature of PBK in lung cancer, we visualized the SCNA and mutation frequencies in the TCGA cohort of 513 LUAD patients and 498 LUSC patients. The DNA mutations used for mapping occurred statistically in 460 (~ 89.7%) LUAD patients and 330 (~ 66.3%) LUSC patients according to the data, and the waterfall plot shows that the overall level of DNA alterations in the PBK gene in lung cancer is about 5% (Fig. 3A, B, D), and we found that the predominant PBK mutation type in lung cancer is Missense_Mutation (Fig. 3C). Differences in mutation frequencies in each group of samples were assessed by chi-square test, and the genes with the highest mutation frequencies with higher PBK expression in LUAD were TP53 (2.0e−5), TTN (2.5e−3), CSMD3 (0.01), RYR2 (4.4e−3), and LRP1B (0.02). In contrast, the situation in LUSC changed to FLG (1.0e−2), MUC5B (7.8e−3), KEAP1 (7.9e−4), EPHA5 (2.2e−3), and STAB2 (0.02) (Fig. 3A, B). Although few samples had mutations in PBK, we observed that mutations in CNV accounted for the majority of PBK gene DNA alterations in all lung cancer patients and found a high proportion of LUAD patients with CNV with multiple tumor suppressor genes, such as CCDC25, SCARA5 and EPHX2 (Fig. 3E). Afterwards, by analyzing the results we could conclude that there was no significant difference in survival between PBK-mut and PBK-wt patients (Fig. 3F). In the pan-cancer TMB correlation analysis correlation, PBK was highly correlated with the TMB of LUAD (Fig. 3G). the alteration of CNV drew our attention, we visualized 23 cancer species containing mutation data and PBK gene expression data, and it is noteworthy that the difference between samples with CNV deletion in LUAD and normal and acquired samples was not significant, but the expression of PBK showed an increasing trend (Fig. 3H). Above, these results suggest that CNV alteration would be a cause of abnormal PBK regulation in LUAD, less related to mutation, and secondly the upregulation of PBK expression is more stable.

### PBK expression affects the clinical features and altered pathway activity of LUAD

**Figure 4:**
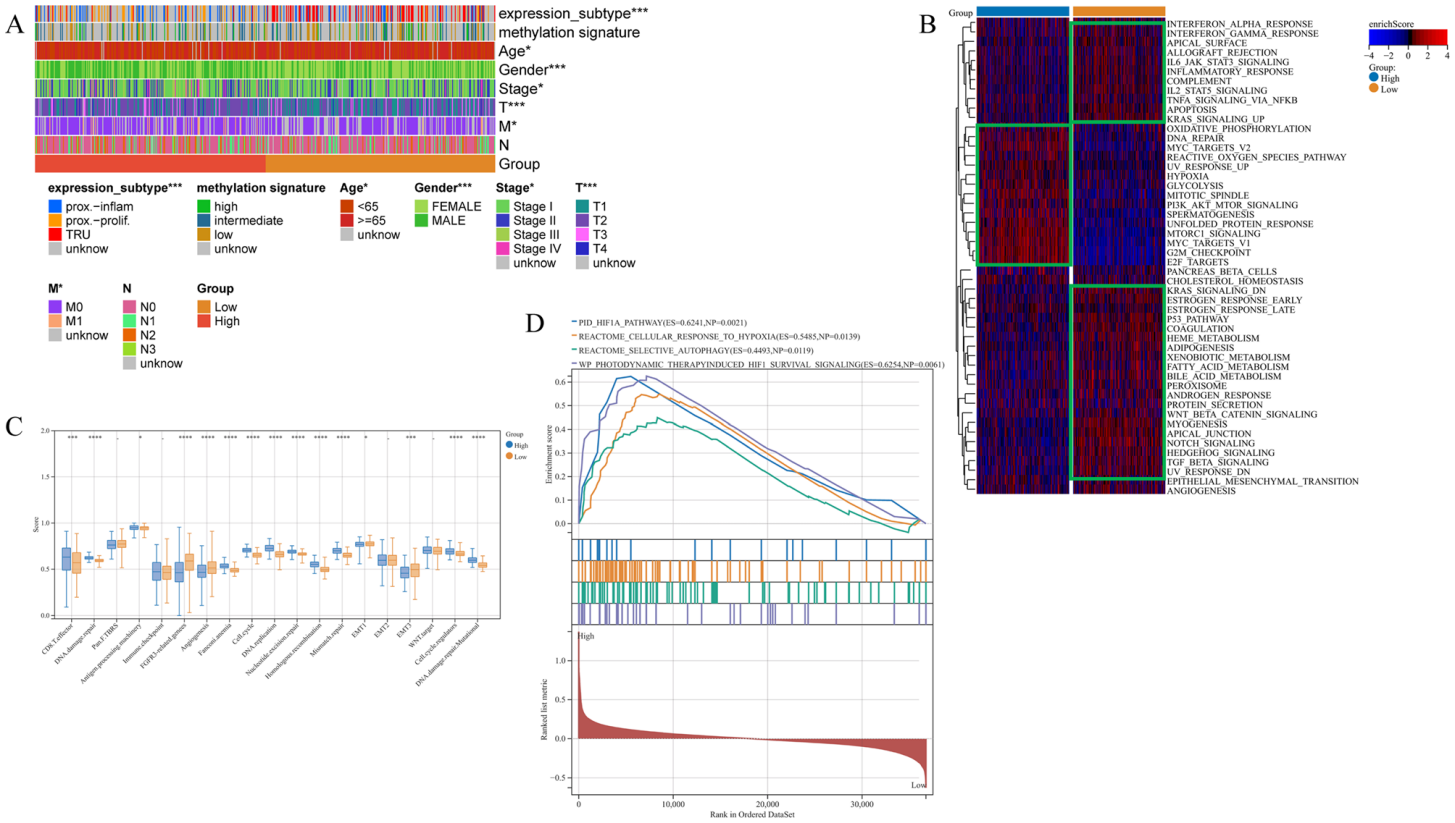
Clinical characteristics and biological course of different PBK expression profiles in LUAD. (**A**) Heatmap of clinical features demonstrating the relationship between high and low expression of PBK in TCGA dataset and clinical features, molecular subtypes of LUAD. (**B**) Heatmap showing the most enriched biological pathways calculated by GSVA algorithm between PBK high and low expression. hallmark gene sets (MSigDB 7.0) were used as reference gene features. (**C**) Angiogenesis, EMT, and other matrix activation features proposed by Mariathasan et al. (**D**) GSEA plots showing HIF1A signaling, hypoxia pathway and autophagy pathway-mediated gene sets enriched in PBK high expression.

The analysis of PBK expression profile we found to be significant in some clinical features and here we explored the relationship between high and low PBK expression in LUAD with conventional clinical features. According to previous studies in which TCGA data were noted to show the overall molecular characteristics of LUAD, the results of the study allow the classification of LUAD into 3 transcriptional subtypes (proximal inflammatory type [PI], proximal proliferative type [PP] and terminal respiratory unit [TRU])^36^. As seen in the heat map plotted containing the combined PBK expression and clinical features, patients with high PBK expression were concentrated in the PP subtype with poor prognosis, whereas the low expression group was concentrated in the TRU subtype with good prognosis (Fig. 4A). High and low expression of PBK caused significantly different distribution in age, sex, staging T and M. We counted and tested the above factors and found that between the high and low expression groups expression_subtype, age, gender, staging T and M. Subsequent forest plots showed that PBK expression could significantly affect patient prognosis compared to other features (Supplementary Fig. 3A, B).

Altered pathway activity usually affects tumor malignant proliferation and progression, and we performed ssGSEA analysis of the Hallmark gene set in LUAD patients (Fig. 4B). The results showed that the PBK low expression group was firstly significantly enriched in immune microenvironment-related pathways, including IFN-γ/α response, IL-6_JAK_STAT3 signaling pathway, inflammatory response, IL2_STAT5 signaling pathway and TNF signaling. Second, we also observed that PBK low expression was highly enriched in tumor metabolism-related pathways, including the HEDGEHOG pathway, and XENOBIOTIC, fatty acids, and BILE ACID METABOLISM. In contrast to PBK low expression, high expression of PBK was mainly associated with oncogenic activation and high proliferative features such as DNA repair, G2_M checkpoint, hypoxia, PI3K/AKT/mTOR, E2F targets and MYC_V1/V2. It is also interesting to note that LUAD was observed to have partial immune activation and immune cell infiltration under PBK high expression, followed by the use of Signaling signature set analysis of Mariathasan et al. tissues showed that CD8 effects and antigen processing mutations were significantly enhanced in the PBK high expression group (Fig. 4C).

To discover the enrichment pathway between PBK high expression and PBK low expression we further investigated using gene set enrichment analysis (GSEA) targeting REACTOME, PID and WikiPathways gene sets. The results revealed that high PBK expression was associated with hypoxia, autophagic process and enhanced HIF signaling (Fig. 4D), while the activation of HIF and the occurrence of autophagy under hypoxia were associated with tumor immune escape. Combined with the poor prognosis of PBK high expression in LUAD patients, we hypothesized that the activation of HIF signaling under PBK high expression leads to immune escape in LUAD patients through hypoxic processes.

### Immune microenvironment analysis reveals the immune infiltration characteristics of PBK in LUAD

**Figure 5:**
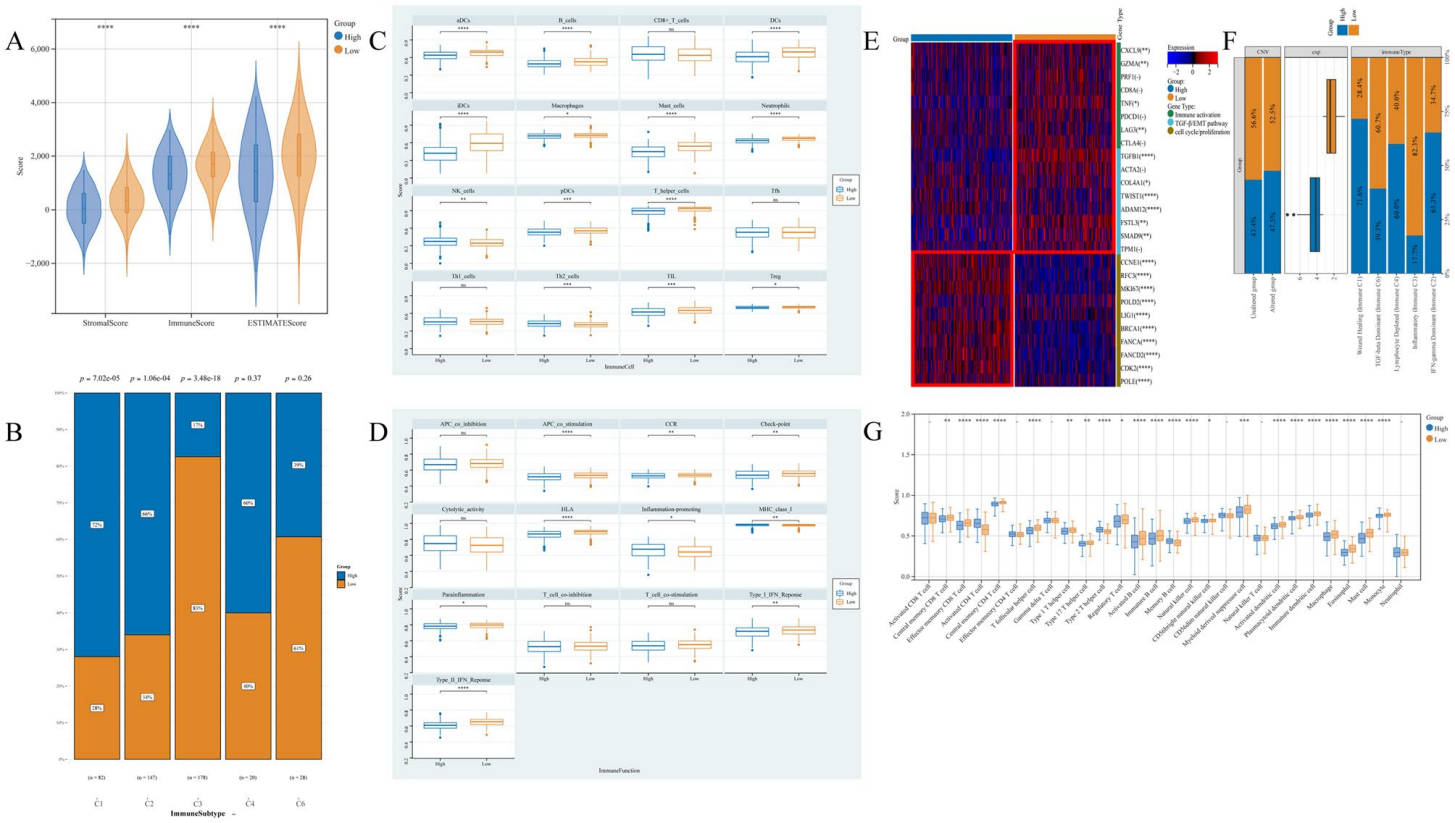
Preliminary exploration of the LUAD immunophenotype and immune cell infiltration between high and low PBK expression. (**A**) Comparison of indicators of immune infiltration levels (both immune score and stromal score) between PBK high and low expression obtained by ESTIMATE using Kruskal–Wallis test. (**B**) Subgroup bar stacked plots showing an overview map of the distribution of immune infiltrative subtypes between PBK high and low expression groups. (**C,D**) The scores of 16 immune cells and 13 immune-related functions were detected by ssGSEA analysis, and the differences between different expression groups of PBK were demonstrated by box line plots. (**E**) Heat map demonstrating the expression of inflammatory, stromal-associated mRNAs in LUAD patients. (**F**) Grouped box line plot containing PBK expression, immune infiltrating subtypes and CNV mutation distribution. (**G**) Demonstration of PBK high and low expression between 28 representative immune cell profiles curated by Charoentong et al.

We further used the ESITIMATE algorithm to quantify the different expression patterns of PBK in the samples, including overall immune infiltration (immunescore) stromal infiltration (stromalscore) and tumor cell purity (ESTIMATE-tumor purity). In LUAD patients with high PBK expression, immune score, stromal score and tumor purity were substantially decreased (Fig. 5A). Previously, immune infiltration of human tumors was classified into six types, namely C1 (wound healing), C2 (INF-G dominant), C3 (inflammation), C4 (lymphocyte depletion), C5 (immune silencing), and C6 (TGF-B dominant). The results showed that in the immune infiltration classification of LUAD patients, PBK high expression group was higher in C1 and C2 types, which were mentioned in other studies as having high proliferative features and associated with Th2 cell bias, M1/M2 macrophage polarization and CD8 signaling, while PBK low expression was higher in C3 inflammatory subtypes, while C3 types had low to moderate tumor cell proliferation and lower levels of altered somatic cell copy number than other subtypes characterized (Fig. 5B, F)^37^.

We first found cellular infiltration in all DC families in 16 immune cell species decreased in the PBK high expression group, which may suggest an impairment of antigen processing and presentation in LUAD patients under PBK high expression, along with a rising trend of CD8+ T cells (Fig. 5C). To better observe the infiltration of immune cells, we used 28 representative immune cells curated by TISIDB for a preliminary validation of the previously observed phenomenon. Similar to the previous results we observed a significant elevation of cytotoxic T lymphocytes and Th2 cells in the PBK high expression subgroup, while regulatory T cells, macrophages, B cells and dendritic cells were reduced (Fig. 5G).

In terms of immune function, we found that compared to tumor versus normal tissue, the PBK high expression group had significantly higher MHC-I class function than the low expression group, while the role of APC_co_stimulation, Check-point, and Parainflammation were all lower than the low expression group in contrast to MHC-I class function (Fig. 5D). We then obtained inflammation-matrix activation-associated mRNAs from LUAD patients mentioned in previous studies to explore the relationship between PBK expression and these with molecularly perturbed environment-related expression (Fig. 5E).

Smoking-induced genomic instability in lung tissue is likewise an important factor contributing to the remodeling of the immune status of LUAD^38^. We observed a significantly higher proportion of the predominantly male smoking population in the PBK high expression group than in the low expression group (Supplementary Fig. 3C), suggesting a strong association between high PBK expression and the male smoking group. Also in the figure, we found that the proportion of STK11 mutations was larger in the high expression group (Supplementary Fig. 3D). Recent evidence from the molecular characterization literature suggests that mutations in selected oncogenic driver genes (e.g., TP53, KEAP1, STK11, KRAS, and EGFR) are associated with an immunosuppressive phenotype in LUAD^39,40^. We therefore plotted the relationship between these gene mutations and high and low PBK expression using bar graphs. It could be more clearly found that the proportion of STK11 mutated tumors in the PBK high expression group was significantly higher than the proportion of unmutated tumors, and in addition to STK11, KEPA1 and TP53 also exhibited this phenomenon, but the difference was not significant (Supplementary Fig. 3E, F).

Based on the above findings, we can speculate that different patterns of PBK expression have different immunophenotypic characteristics; the anti-tumor cells and functions are increased to different degrees in these LUAD patients with high PBK expression, but the anti-tumor environment in LUAD patients is still poor, and we speculate that the high PBK expression is more inclined to an immune rejection phenotype, which is specific, with high anti-tumor immune cell infiltration but significantly impaired antigen presentation and activation. Low PBK expression tends to be a normal immune phenotype, characterized by an activated immune pathway and a high infiltration of immune cells.

### LUAD tumors with high expression of PBK showed significant immune escape

**Figure 6:**
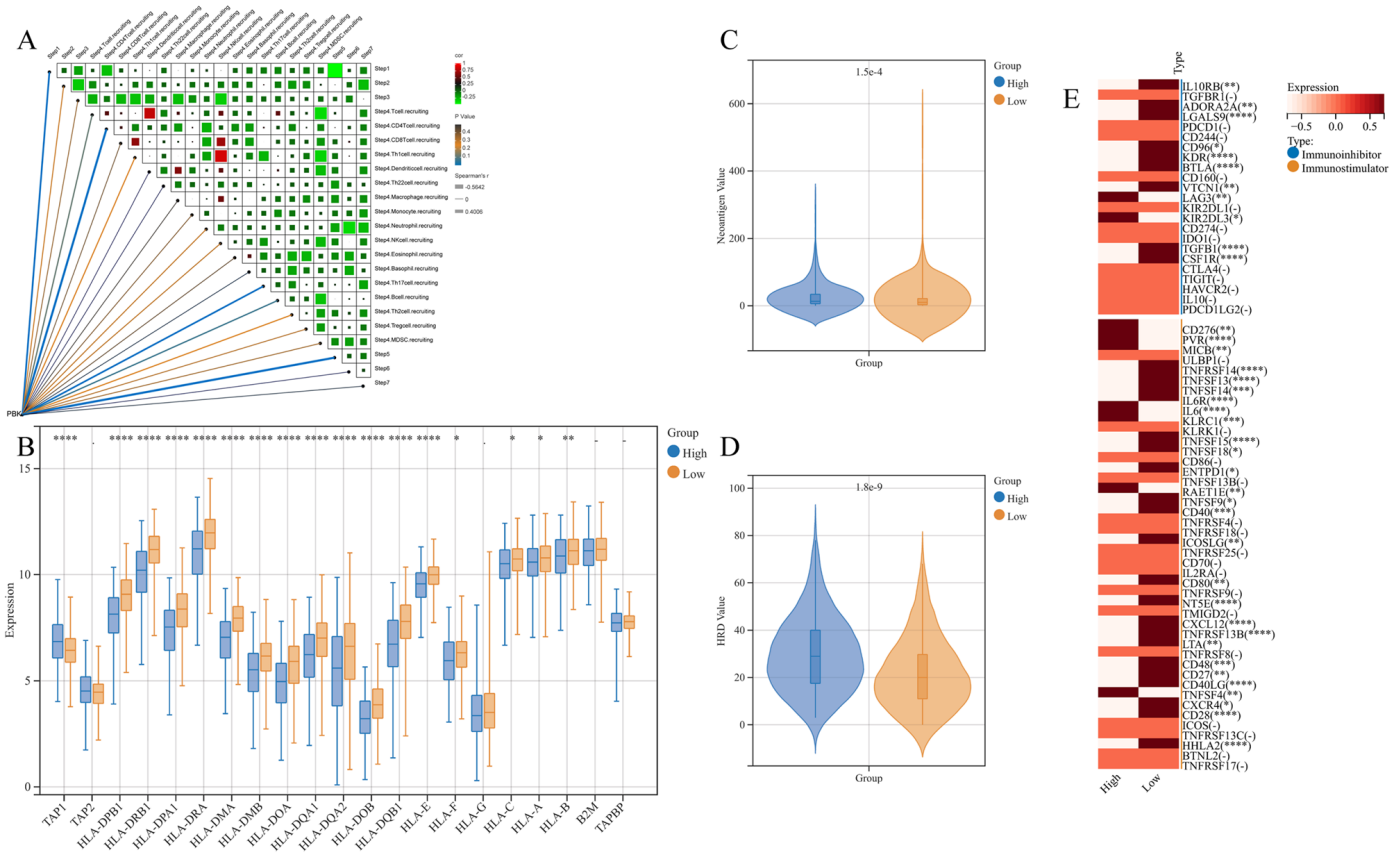
(**A**) Butterfly correlation plot showing the correlation analysis between PBK gene expression and 7-step tumor immune cycle. (**B**) Expression of HLA and genes associated with MHC molecules between PBK high and low expression groups. (**C,D**) Differences in neoantigen load and HRD levels between PBK high and low expression groups. (**E**) Relative expression levels of immunosuppressive and immunostimulatory agents in PBK high and low expression groups. ***P < 0.001; **0.001 < P < 0.01; *0.01 < P < 0.05; –: P > 0.05.

The causes of immune escape in tumors as indicated by previous studies can be broadly explained in the following ways (1) defective antigen presentation in the tumor microenvironment; (2) tolerance and immune bias of the organism; (3) immunosuppressive cell infiltration; (4) alteration of immunoregulatory genes (41). The next tumor immune cycle correlation analysis pointed out that PBK is most likely associated with cancer cell antigen release (step 1), immune cell transport to the tumor (step 4), and immune cell infiltration into the tumor (step 5) (Fig. 6A). We will propose to start from the discovery of the effect of PBK high expression on tumor antigen and immune cell infiltration.

### Cancer cell antigens and immune tolerance, bias

The factors of defective tumor antigen presentation may include two aspects, firstly, changes in tumor immunogenicity, such as the emergence of neoantigens, and down-regulation or absence of antigen presentation pathways in the organism. Here we focused on for the assessment of tumor immunogenicity we chose HRD and Neoantigens metrics, with high PBK expression having the highest neoantigen load and HRD score (Fig. 6C, D). For the expression of antigen presentation pathway-related genes as shown in the box plot, the PBK high expression group showed decreased expression in most MHC-related genes, while the low expression group showed elevated expression (Fig. 6B). Overall, there was some degree of impaired antigen presentation and the most pronounced decrease in immunogenicity and antigen-presenting gene expression levels under PBK high expression. It has been previously reported that tumor-induced immune tolerance in the organism can be achieved by modulating immunostimulatory molecules. The relative expression levels of immunosuppressive and immunostimulatory agents are shown in the figure. We labeled the genes with significant and insignificant expression between the two groups. Low expression of PBK had a better anti-tumor immune activation phenomenon, which was demonstrated by the highest expression levels of most immunosuppressants and some immunostimulants. In contrast to PBK low expression, we found that the activation levels of most of the above mentioned genes were lowest in the PBK high expression group (Fig. 6E), then the decreased anti-tumor activity in the high expression group would be replaced by a pro-tumorigenic one. Overall, PBK high expression was accompanied by reduced expression of most immunostimulants and immunosuppressive agents and poor immune tolerance, while PBK low expression had the best expression levels and low tolerance to immune activity.

### Disturbed anti-tumor immune response

**Figure 7:**
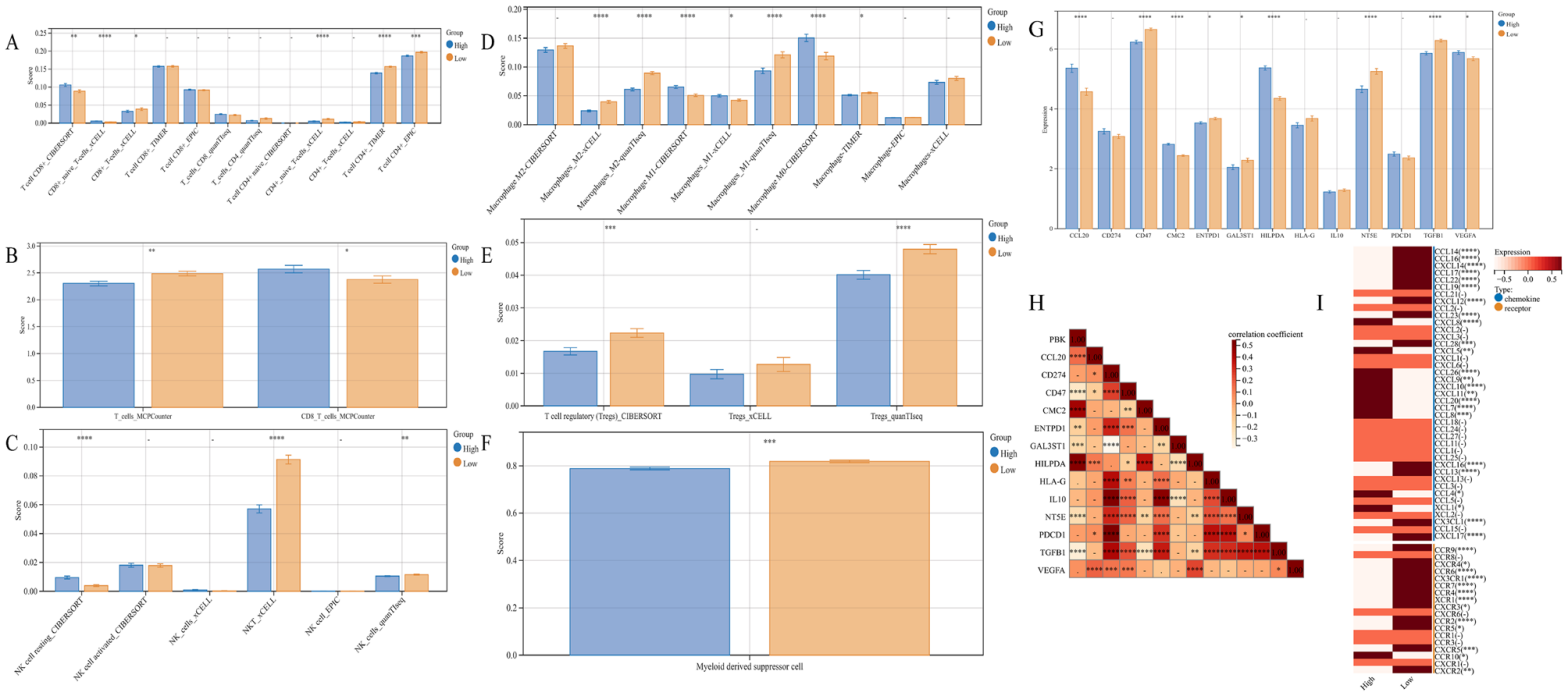
(**A–C**) Infiltration distribution of T cell subpopulations and NK cell subpopulations associated with antitumor immunity in PBK high and low table groups. (**D–F**) Infiltration distribution of Macrophages subpopulations, Treg cells and MDSC associated with immunosuppression in PBK high and low table groups. (**G,H**) Expression levels of some genes associated with tumor immune escape and correlation analysis. (**I**) Relative expression levels of chemokines and receptors in PBK high and low expression groups. ***P < 0.001; **0.001 < P < 0.01; *0.01 < P < 0.05; –: P > 0.05.

To more comprehensively observe the infiltration pattern and immune response of immune cells in the organism of LUAD patients, we introduced xCELL, TIMER, CIBERSORT, MCPCounter, EPIC and quanTIseq immune infiltration algorithms for a comprehensive analysis of LUAD. All immune cell infiltration and immune-related chemokines, receptors, MHC and immunostimulatory, and inhibitory molecules we show the results/expression in the heat map (Supplementary Fig.4A, B). We first determined the activation CD4 and CD8 T cell infiltration and it is evident that PBK high expression has a higher activation CD8 T cell infiltration but the overall number of this cell type did not increase, followed by CD4 T cells showing activation and an overall decrease in the level (Fig. 7A, B). the NK cell population is similar to the CD8+ T cell situation, with the total number of NK cells showing upward trend, while NK Tcell decreased substantially (Fig. 7C). tregs, MDSCs and M2 macrophages as immunosuppressive cells we observed a decrease in all these immunosuppressive cells under high PBK expression (Fig. 7D–F). Furthermore, correlation analysis of immune cell infiltration in TCGA dataset and GEO dataset revealed that PBK expression strongly correlated with dendritic cells and Th2 cells, but infiltration of dendritic cell family was decreased in all PBK patients under high expression (Supplementary Fig. 4A–D). It is clear that PBK overexpression affects T cell activation, downregulation of MHCI-like genes and thus leads to reduced type 1 and 2 interferon γ responses, which prevent T cells from playing their anti-tumor role properly and tumors undergo significant immune escape.

We then compared PBK high and low expression of chemokines and receptors. In the PBK high expression group, the expression levels of pro-immune chemokines such as CXCL9, CXCL10 and CXCL11 were higher (Fig. 7I), and these immune chemokines can function to recruit effector T cells and NK cells^42^. However, IDO1, which contributes to peripheral tolerance, was upregulated but not significant in PBK, suggesting the possible presence of cytokines in the suppressive inflammatory microenvironment (Supplementary Fig. 4E). It has been shown that the tumor-derived cytokine CCL20 upregulates IDO expression, and IDO also inhibits CD8+ T cell responses and induces tumor immune evasion, so it is highly likely that the strong rise of CCL20 in PBK overexpression inhibits CD8+ T cells via IDO1 (Fig. 7G). Secondly, the CCL20-CCR6 axis was reported to primarily affect chemoattraction to immature dendritic cells (DCs), effector/memory T cells and B cells in the tumor immune microenvironment^43^, and the decrease of CCR6 in the PBK high expression group caused a decrease in DC infiltration which is consistent with the above results (Fig. 7I). Notably, significantly higher levels of VEGFA were expressed under PBK high expression. also under the condition that PBK is associated with hypoxia we will summarize the mRNA levels of downstream targets associated with HIF-1 activation and immune checkpoint molecules and analysis of genetic correlation with PBK that could potentially promote immune escape of cancer cells (Fig. 7H).

### Identification of genes differing between groups with high and low PBK expression and analysis of biological processes

**Figure 8:**
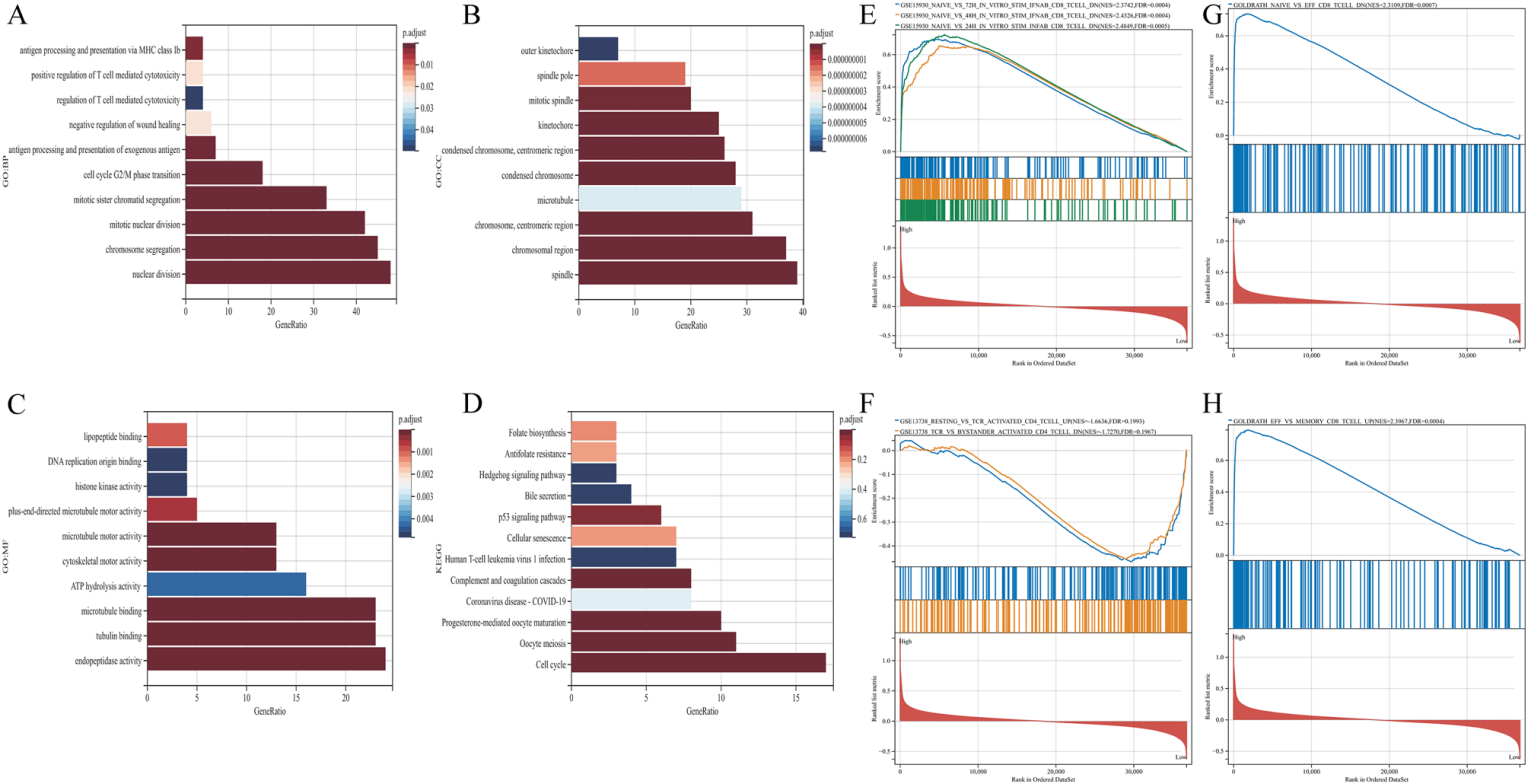
Gene enrichment analysis of differential genes between PBK high and low expression groups. (**A–C**) Bars show the enrichment results of GO:BP, GO:CC and GO:MF, in that order. (**D**) The most significant pathway in KEGG. (**E–H**) GSEA analysis between groups with high and low PBK expression.

We first grouped LUAD patients by median PBK expression and identified 353 differential genes from different expression groups of LUAD according to the criteria of |logFC |> 1.5, adjusted P value < 0.05. And these differential genes were further investigated for their biological significance. In Fig. 6A, differential genes were mainly associated with malignancy proliferation (cell cycle G2/M phase transition, p53 signaling pathway and cell cycle) and immune cell function (positive regulation of T cell-mediated cytotoxicity, regulation of T cell-mediated cytotoxicity and cell cycle) according to GO and KEGG annotations. regulation of T cell-mediated cytotoxicity) and immune cell function (positive regulation of T cell-mediated cytotoxicity, regulation of T cell-mediated cytotoxicity) have a clear interaction, followed by involvement in antigen processing and presentation via MHC class Ib, and negative regulation of wound healing and exogenous antigen processing and presentation (Fig. 8A–D). Overall, genes between the high and low PBK expression groups may have a greater role in immune function, and cancer progression in LUAD.

To further clarify the immune pathway alterations brought about between the high and low PBK expression groups, using the C7 gene set (c7.immunesigdb.v2023.2.Hs.symbols) in the GSEA database we further evaluated the differential genes. The results showed that the PBK high expression scenario had enrichment with the CD8-IFN descent pathway(Fig. 8E), whereas the PBK low expression was enriched for TCR signaling associated with CD4 T cells (Fig. 8F), and interestingly we also observed a downregulation of the effector CD8 cell pathway, upregulation of the memory CD8 T cell pathway, and upregulation of the Naive CD8 T cell pathway in the high PBK expression (Fig. 8G, H). These results also coincide with our above studies on the part of immune escape that may be induced by high PBK expression.

### PBK-related genes construct prognostic models and predictive Nomogram to predict prognosis of LUAD patients

**Figure 9:**
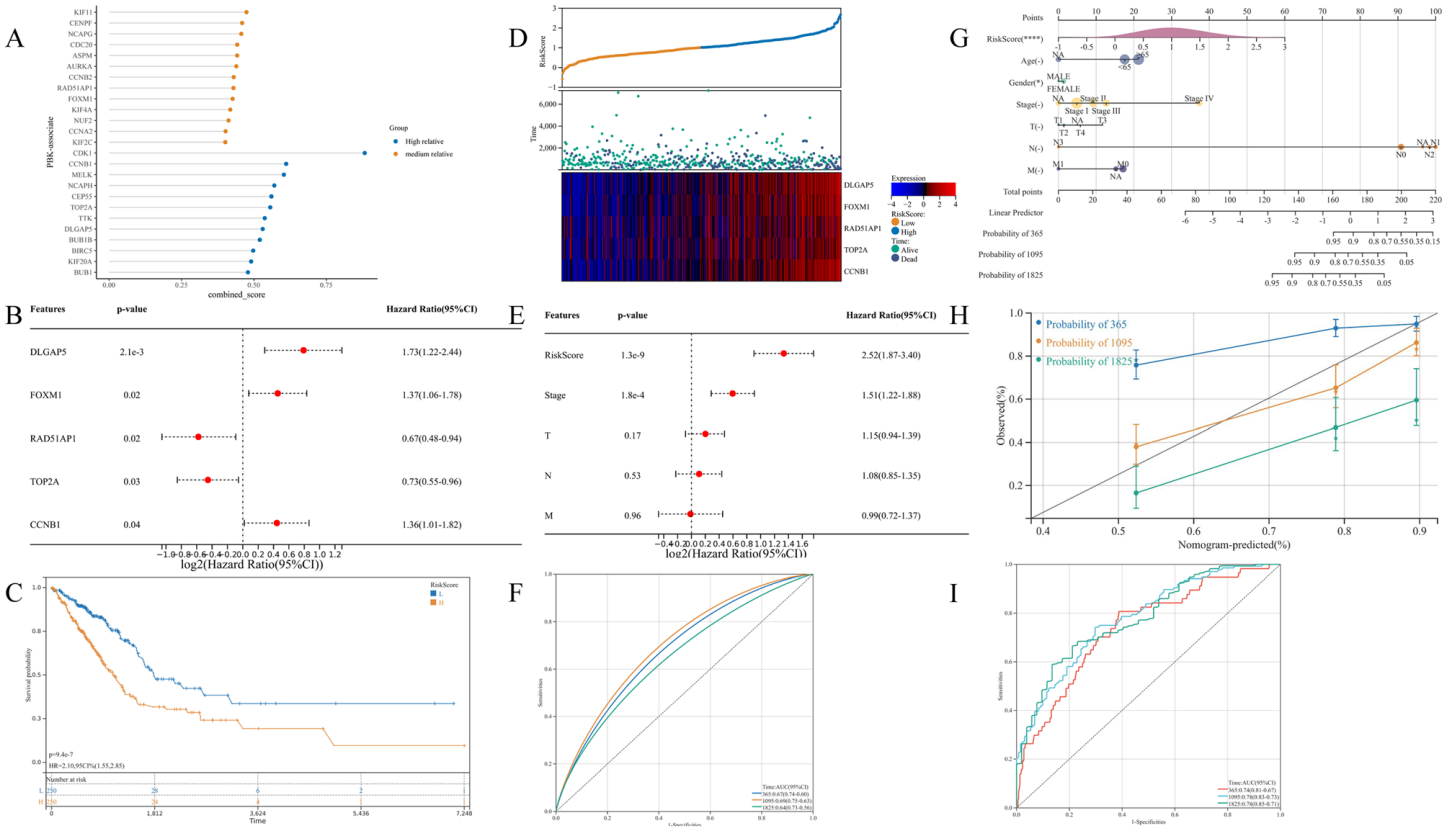
PBK-derived gene modeling for predicting prognosis of LUAD patients. (**A**) String median confidence level screening of genes most associated with PBK from DEG. (**B**) Multifactorial COX analysis yielded 5 genes associated with prognosis of LUAD. (**C**) Kaplan–Meier plots for high and low risk groups. (**D**) Prognostic heat map showing risk score, survival status and gene expression distribution. (**E**) Multifactorial COX results of clinical characteristics combined with risk scores. (**F**) ROC curves demonstrating the probability of OS at 1, 3 and 5 years for risk scores. (**G,H**) Estimation of 1, 3and 5-year survival probabilities by integrating independent prognostic indicators (risk score, age, sex, Stage, T, N, and M for the PBK genome) to utilize prognostic nomogram. Calibration plots show the association of predicted OS with actual survival. (**I**) The predictive efficacy of this nomogram is validated by ROC curves for 1, 3and 5 year survival.

To identify 353 differential genes between high and low PBK expression groups with prognostic associations in LUAD, we performed univariate COX regression and obtained 201 differential genes significantly associated with prognosis in LUAD patients. By String database analysis, sorted by PBK median confidence, we obtained the most prognostic genes associated with PBK, (Fig. 9A). Based on a median confidence level > 0.4, 25 characteristic PBK-derived genes were identified (Supplementary Table 1). Based on this, we performed multivariate Cox regression models to obtain a prognostic model for PBK-associated genes. The results showed that the risk score = 0.250901594 * CCNB1 expression + (−0.446288847) * TOP2A expression + 0.436757566 * DLGAP5 expression + (−0.400801728) * RAD51AP1 expression + 0.197773942 * FOXM1 expression (Fig. 9B). Grouping according to the median risk score, we classified LUAD patients into high-risk and low-risk subgroups. Survival analysis showed that high-risk LUAD patients exhibited a poorer prognostic profile. A higher number of patients with death in the high-risk subgroup could be observed from the prognostic heat map (Fig. 9C, D). In addition, the gene expression of CCNB1, TOP2A, DLGAP5, RAD51AP1 and FOXM1, which comprise the prognostic model, showed significant differences in the two subgroups, and the KM curves showed that the high expression of these genes responded to the worsening prognostic status of LUAD patients (Supplementary Fig. 5A–E). The results returned from the ROC curves showed that the prognostic model constructed from PBK-related genes was more stable in estimating the probability of overall survival at 1, 3 and 5 years (Fig. 9F).

To determine the independent effect of PBK risk score in prognosis, we introduced prognostic analysis of T, N, M and staging clinical characteristics based on risk score by multifactorial Cox regression, and the results showed that the risk scores of staging and PBK-related genes could be used as independent prognostic indicators for LUAD (Fig. 9E). To assess the survival outcome of LUAD patients, we combined the risk scores and clinical information of LUAD patients to draw Nomogram, and the results are shown in Fig. (Fig. 9G). Compared to other clinical features, we found that risk scores for PBK-related genes were the most meaningful in predicting survival time in LUAD patients. This observation can be further explained from the calibration curves. And the predicted results of Nomogram for LUAD patients at 1, 3 and 5 years were also closer to the actual survival (Fig. 9H). The final ROC curve validation results likewise confirmed the expected role of Nomogram in predicting survival outcomes in LUAD patients (Fig. 9I). Similarly, based on the same formula and analytical approach we validated two external cohorts, GSE37745 and GSE72094.The KM analysis showed that the results were consistent with those of the TCGA cohort(Supplementary Fig. 5F, I), and the ROC results were more stable(Supplementary Fig. 5G, J). In addition, multivariate Cox analysis of the two cohorts showed that risk score was also an independent factor(Supplementary Fig. 5H, K). Based on these results, it can be concluded that the 5-gene prognostic model associated by PBK is more stable and the Nomogram also has good predictive ability.

### Drug sensitivity analysis for PBK and PBK-related gene models to guide LUAD chemotherapy strategy

**Figure 10:**
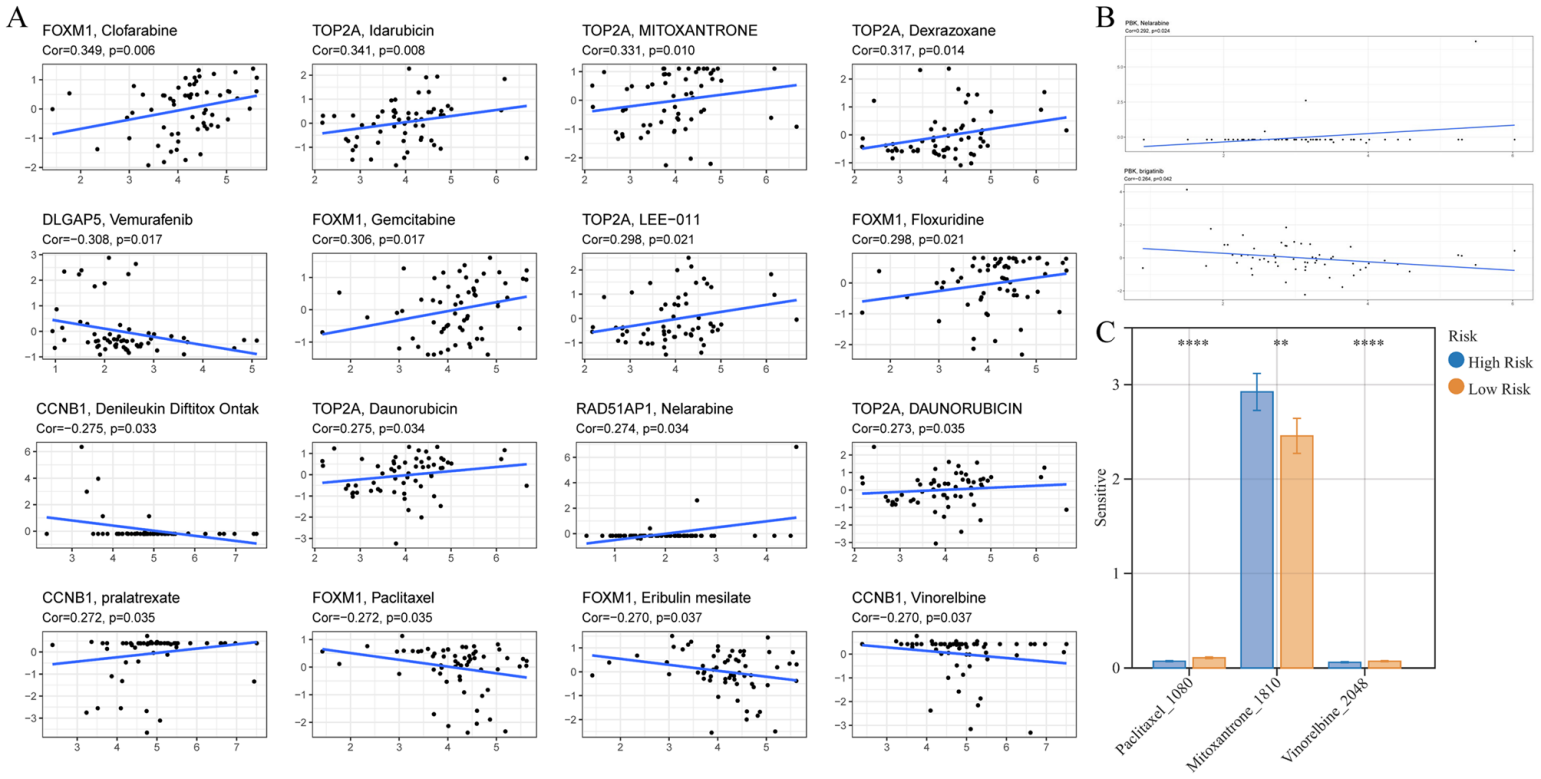
PBK and PBK-derived genomic models to predict drug response in LUAD patients. (**A**) Correlation diagram of genes comprising the PBK-associated gene model with small molecular compounds in LUAD. (**B**) PBK gene correlation analysis with nelarabine and brigatinib. (**C**) Levels of drug sensitivity in risk groups at Mitoxantrone_1810, Paclitaxel_1080 and Vinorelbine_2048.

Tumor cell death induced by certain chemotherapeutic agents in clinical regimens can be used to amplify tumor sensitivity to drugs in tumors that are resistant to checkpoint therapy, so the optimal combination of chemotherapy and immunotherapy deserves further exploration^44^. We found that the PBK gene was only positively correlated with nelarabine (Cor = 0.292, p = 0.024) and negatively correlated with brigitinib (Cor = -0.264, p = 0.042) in drug prediction (Fig. 10B). Subsequent in-depth studies on the action of PBK with the above two drugs may provide new reference values for chemotherapy regimens in lung adenocarcinoma patients. Since PBK-associated gene model scores were generated based on prognosis-related DEGs between high and low PBK expression, we speculate that chemotherapy status may correlate with the level of PBK-associated gene model scores. Using the OncoPredict package to predict drug sensitivity for high and low PBK-associated gene model risk group scores, we demonstrated that the three significant drugs of Mitoxantrone_1810 were positively associated with Risk, while Paclitaxel_1080 and Vinorelbine_2048 were negatively associated (Fig. 10C). We also evaluated the interaction of genes comprising the PBK-related gene model with drug response. As a result, FOXM1 was positively and negatively correlated with Clofarabine, Gemcitabine, Floxuridine Paclitaxel and Eribulin mesilate; TOP2A was positively and negatively correlated with Idarubicin, MITOXANTRONE, LEE-011 Daunorubicin and DAUNORUBICIN. DLGAP5 was negatively associated with Vemurafenib, and CCNB1 was negatively associated with Denileukin Diftitox Ontak, Vinorelbine but positively associated with pralatrexate (Fig. 10A). These data suggest that PBK-related genes may be associated with the sensitivity of the above mentioned drugs.

## Discussion

This study presents a comprehensive analysis of the molecular and clinical features, immune status and genomic drug profile of the LUAD subtype of lung tumors in particular, starting from the basis of PBK expression and prognosis in pan-cancer. Our results suggest that PBK is upregulated in the vast majority of tumors, while mutations in the PBK gene affect some of the oncogenes of LUAD but not their own upregulation, while the gene is more stably altered in LUAD^45^. Previous reports have shown that lung cancer cells can directly release angiogenic factors, such as VEGF, that stimulate the formation of neointima in the tumor and extra-tumor stroma^46^. And PBK may regulate tumorigenesis, progression and immune status by affecting hypoxic processes and HIF signaling pathway. In the present study, we found that GSEA analysis of PBK high expression group showed that PBK was associated with hypoxic process and HIF pathway. This finding is consistent with previous reports.

Exploring the links between the immune microenvironment and tumor immune status during tumorigenesis and progression will provide a comprehensive understanding of tumor molecules and their biological characteristics, and provide potential markers for prognostic assessment and treatment options for LUAD^38^. We used a large number of clinical and molecular features to demonstrate the degree of conformity of different PBK expression to the immune phenotype, and finally obtained that high PBK expression tends to the immune rejection phenotype. This immunophenotype is specific in patients with high PBK expression, and a large number of algorithms and data validate that anti-tumor immune cell infiltration is increased but the patient's immune profile remains poor, and secondly that immunosuppressive cells do not play a strong role in the tumor microenvironment. However, on the other hand, PBK was strongly positively correlated with CCL20, CMC2 and HILPDA, and CCL20 was reported to upregulate indoleamine 2,3-dioxygenase (IDO) expression through the IFN-γ signaling pathway, causing IDO to suppress CD8+ T cell responses and induce tumor immune evasion, and previous studies observed that immunosuppressive factors IDO and PD-L1 in the organism high expression levels in metastatic melanoma CD8+ T cells appeared over^47^. In the last part, CCL20 and CCR6 can form a chemokine regulatory axis, which in turn regulates the interaction between cancer cells and immune cells, thus influencing the immune status of the tumor in the state of the tumor immune microenvironment and affecting the immune system against the tumor^48^. High PBK expression affects the downregulation of CCR6 preventing cells such as effector/memory T cells and DCs from being attracted into the tumor for further tumor immune escape.

The alteration of related genes caused by PBK gene expression is another aspect that affects the tumor biology of LUAD patients, the functional analysis of differential genes between PBK high and low expression groups showed that these genes play a role in malignant tumor proliferation, such as: cell cycle G2/M phase transition and p53 signaling pathway, while for T cell function we found to the positive regulation of T cell-mediated cytotoxicity and processes such as regulation of T cell-mediated cytotoxicity, all these results suggest that differential genes play an important role in the progression and immune status of LUAD. Preliminary analysis confirmed that the prognostic differential genomic model associated with PBK genes is reliable and can independently predict patient prognosis and relapse, and can be validated on multiple external datasets in the future. Future validation on multiple external datasets of the new LUAD will further establish the reliability of the model. We also performed drug prediction on PBK gene and PBK-related genomic models. Correlation analysis showed that PBK was associated with neralide and brigatinib, and in the related genome with clofarabine, gemcitabine, fluorouracil, paclitaxel, and other drugs. The nomogram is a powerful tool based on multifactorial regression analysis that integrates multiple predictors to quantify the risk of each sample in the clinical factors^49^ according to the degree of contribution of each influencing factor in the model to the outcome variable (magnitude of the regression coefficient). In our study by combining PBK-related genomic model risk scores, stage, T, N and M clinical characteristics of LUAD patients, the Nomogram was used to predict risk scores against 1, 3 and 5 years survival time. Subsequently, ROC curves showed that bar graphs showed good efficiency in predicting OS outcomes for individual patients. In addition, the calibration curves confirmed that the actual survival time was consistent with the survival time estimated by the nomogram.

Our study also has some limitations. the expression of PBK needs to be validated on basic experiments and secondly multiple immunotherapy cohort datasets should be introduced for critical validation of the immune status of PBK affecting LUAD and PBK-related genomic models. In addition, the role and mechanisms of PBK in tumor immunity need to be validated by designing a complete experimental program. Finally, the prognostic value of PBK needs to be validated in a future, more comprehensive LUAD cohort.

## Conclusion

Overall, our study utilized a combination of pan-cancer and single cancer species, and we aimed to reveal the clinical features of the impact of PBK in LUAD, associated immune infiltration features, staging and immune escape profiles, and pharmacogenomic profiles of immunotherapy and chemotherapy. In conclusion, our findings suggest that PBK is a potential prognostic marker promoting immune escape in patients with LUAD and a predictor of pharmacotherapeutic response.

### Supplementary Information

Supplementary Information.

## Data Availability

This study analyzed the current publicly available dataset. The data analyzed above can be found here: https://portal.gdc.cancer.gov/, https://www.cbioportal.org/, https://cptac-data-portal.georgetown.edu/cptac. Parts of the public data set are also available from the corresponding author(lianchaoqun@bbmc.edu.cn).

## Abbreviations

PBK: PDZ-binding kinase
NSCLC: Non-small cell lung cancer
TCGA: The Cancer Genome Atlas
TME: Tumor microenvironment
OS: Overall survival
DFS: Disease-free survival
PFS: Progression free survival
NCI-60: National Cancer Institute
ACC: Adrenocortical carcinoma
BLCA: Bladder urothelial carcinoma
BRCA: Breast invasive carcinoma
CESC: Cervical squamous cell carcinoma and endocervical adenocarcinoma
COAD: Colon adenocarcinoma
READ: Colon adenocarcinoma
ESCA: Esophageal carcinoma
GBM: Glioblastoma multiforme
HNSC: Head and neck squamous cell carcinoma
KICH: Kidney chromophobe
KIRC: Kidney renal clear cell carcinoma
KIRP: Kidney renal papillary cell carcinoma
LAML: Acute myeloid leukemia
LGG: Brain lower grade glioma
LIHC: Liver hepatocellular carcinoma
LUAD: Lung adenocarcinoma
LUSC: Lung squamous cell carcinoma
MESO: Mesothelioma
OV: Ovarian serous cystadenocarcinoma
PAAD: Pancreatic adenocarcinoma
PCPG: Pheochromocytoma and paraganglioma
PRAD: Prostate adenocarcinoma
READ: Rectum adenocarcinoma
STAD: Stomach adenocarcinoma
TGCT: Testicular germ cell tumors
THCA: Thyroid carcinoma
UCEC: Uterine corpus endometrial carcinoma
UCS: Uterine carcinosarcoma
