# Supplementary material for: PBK correlates with prognosis, immune escape and drug response in LUAD
Source: Sci Rep. 2023 Nov 22;13:20452. doi: 10.1038/s41598-023-47781-7 (PMC10665404; doi:10.1038/s41598-023-47781-7)
Supplement: Supplementary file 1 — Supplementary Information. [file 41598_2023_47781_MOESM1_ESM.pdf]

Supplemental Information

PBK correlates with prognosis, immune escape and drug response in LUAD

Hongyu Ma, Jing Zhang, Yan Shi, Ziqiang Wang, Wenhu Nie, Xiaojing Wang, Chaoqun Lian

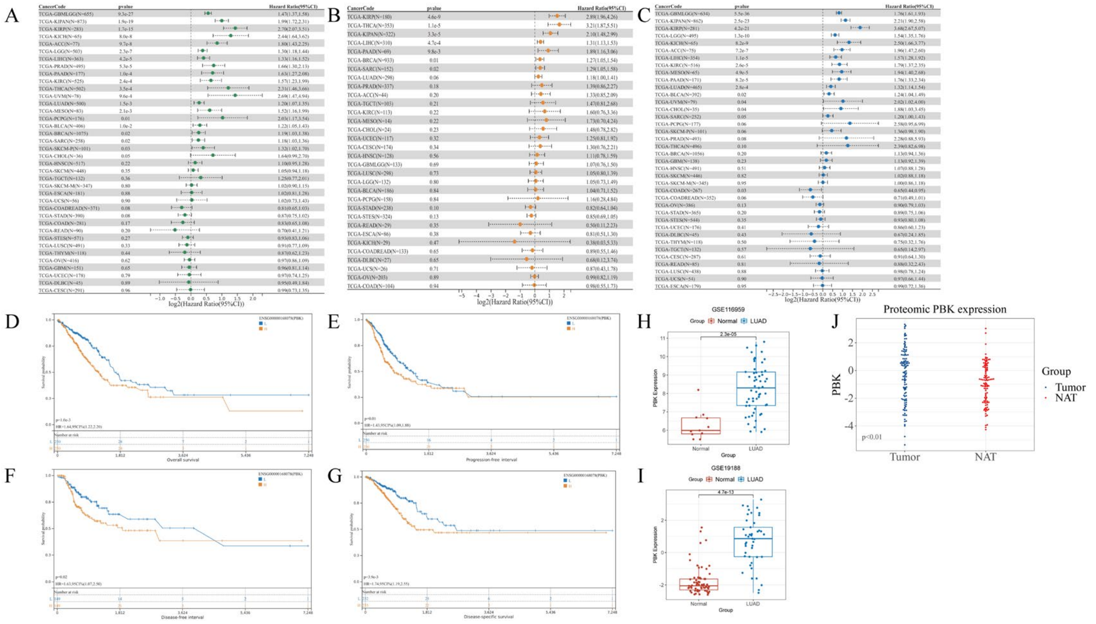

Supplementary Figure 1: Prognostic profile of PBK in OS, PFS, DFS and DSS in pan-cancer analysis and validation of PBK at mRNA and protein levels. (A-C)Prognostic correlation between PBK expression and Progression-free survival (green),Disease-free survival(yellow) and Disease-specific survival (blue)in pan-cancer. (D-G)Kaplan-Meier curves showing survival of patients in the high and low PBK expression groups for Overall survival, Progression-free survival, Disease-free survival, and Disease-specific survival. (H-J) Validation of PBK at protein expression level and GSE116959、GSE19188 mRNA expression level.

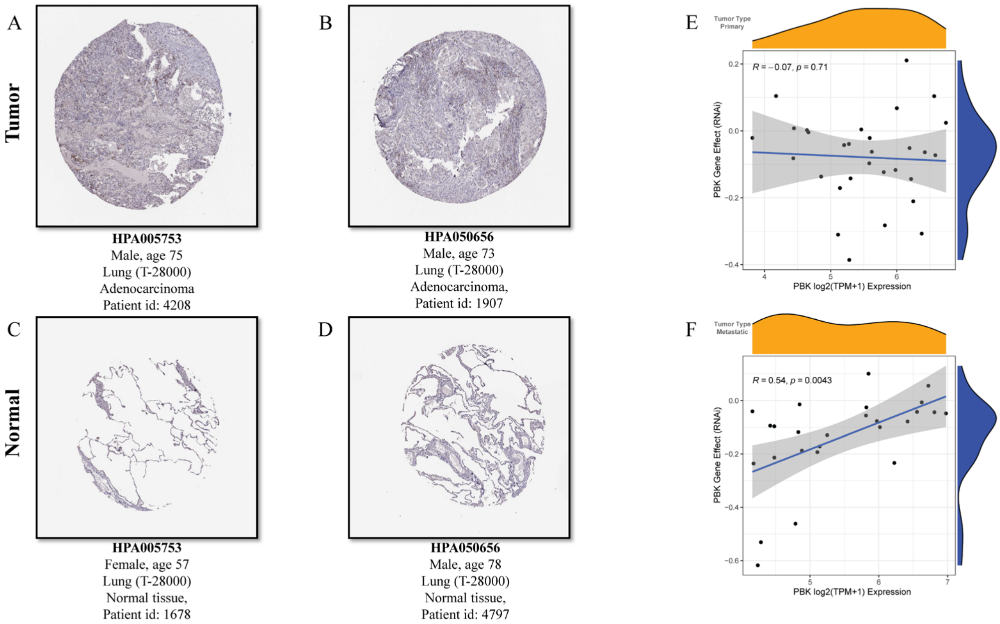

Supplementary Figure 2:External experimental data to validate the PBK protein expression profile and the correlation between PBK gene effects and expression in cell lines after RNAi .(A、B) Derived from immunohistochemical staining data of tissue sections from LUAD patients in the HPA database.(C、D) HE staining results of normal lung tissue sections from the HPA database.(E、F) Correlation of cell line gene effects induced by PBK gene after RNAi with PBK gene expression provided by Depmap database.

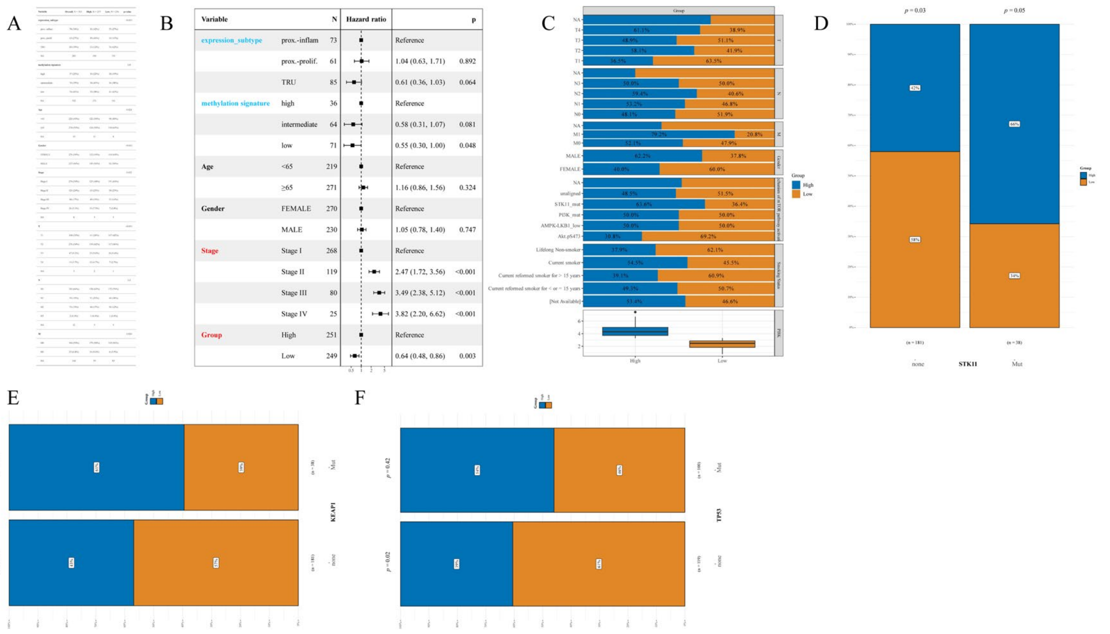

Supplementary Figure 3: Clinical and molecular characteristics between different PBK expression groups. (A) Distribution and Chi-squared test of molecular and clinical features in LUAD between two groups of PBK expression.(B) Forest plots of important molecular and clinical features from LUAD. (C) Multiple groups showing the distribution of smoking factors among different PBK expression groups in mutation, sex, T, N and M. (D-F) Grouped histograms showing the percentage of mutations in SKT11, KEPA1 and TP53 genes in PBK high and low expression groups.

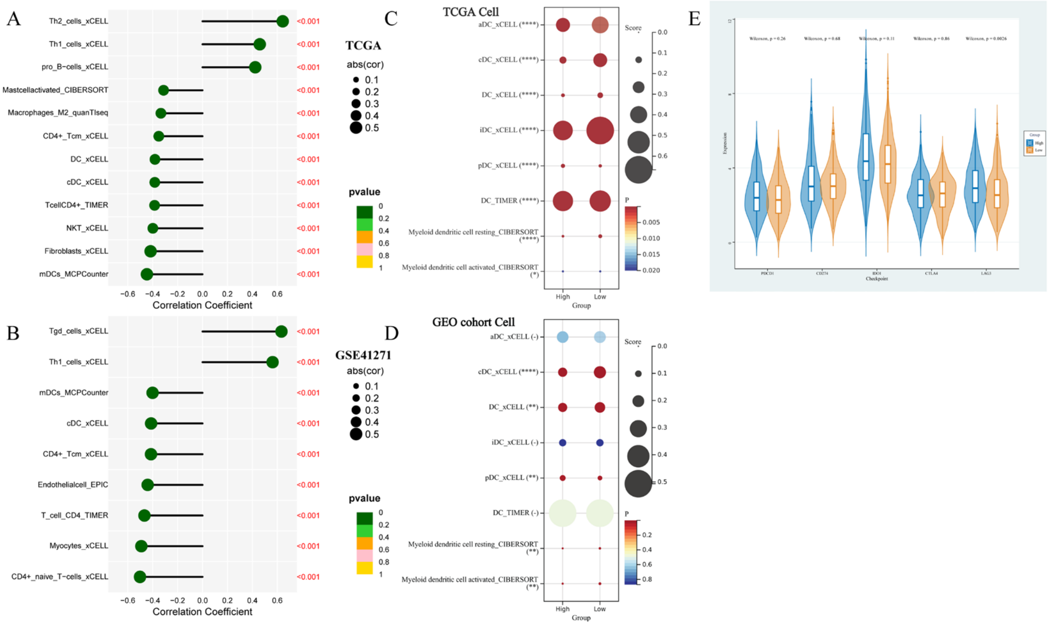

Supplementary Figure 4: Correlation analysis of PBK with immune cell correlation, dendritic cells and immune checkpoints. (A、C) Correlation between PBK expression levels and the abundance of immune cell infiltration in the TCGA cohort. (B、D) Correlation between PBK expression levels and the abundance of immune cell infiltration in the GEO cohort.(E) Levels of immune checkpoint expression between different PBK expression groups.

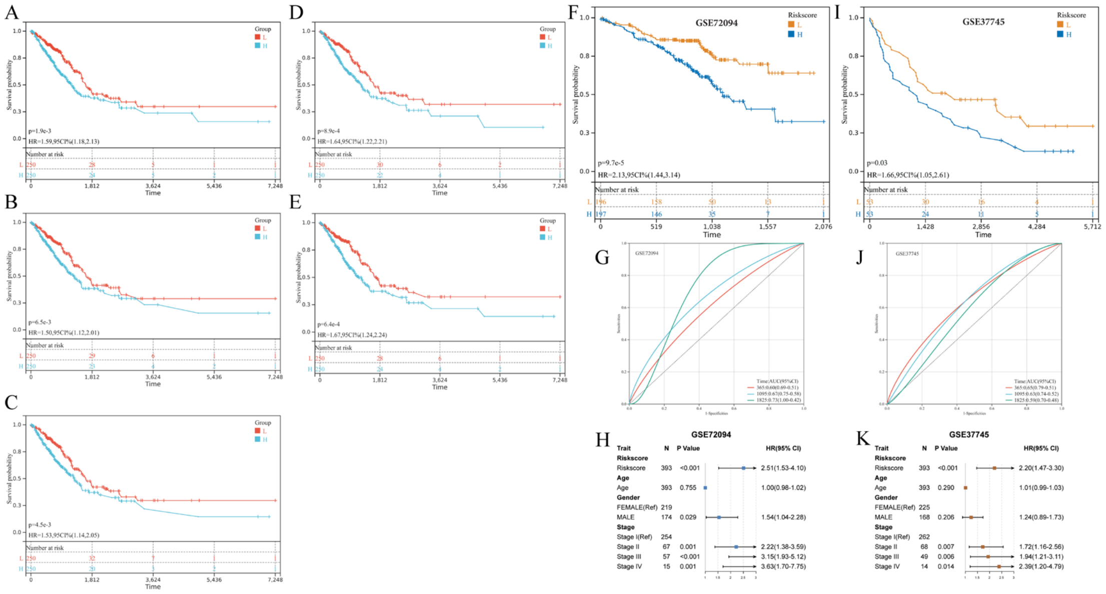

Supplementary Figure 5: Kaplan-Meier plot of the 5-item gene model of the PBK-derived genome obtained by multifactorial COX and validation of the external data set. (A-D) FOXM1, CCNB1, TOPA2, RAD51AP1 and DLGAP5 in that order.(F-H) Kaplan-Meier curves, ROC curves, and multifactorial COX regression forest plots for the 5-gene prognostic model of the GSE72094 cohort.(I-K) Kaplan-Meier curves, ROC curves, and multifactorial COX regression forest plots for the 5-gene prognostic model of the GSE37745 cohort.

Supplementary Table 1

PBK-related derived gene correlation confidence

PBK_related derived gene	combined_score	Group_Median(0.474)
CDK1	0.882	High relative
CCNB1	0.611	High relative
MELK	0.603	High relative
NCAPH	0.57	High relative
CEP55	0.56	High relative
TOP2A	0.556	High relative
TTK	0.537	High relative
DLGAP5	0.53	High relative
BUB1B	0.52	High relative
BIRC5	0.497	High relative
KIF20A	0.49	High relative
BUB1	0.479	High relative
KIF11	0.474	medium relative
CENPF	0.459	medium relative
NCAPG	0.456	medium relative
ASPM	0.442	medium relative
CDC20	0.442	medium relative
AURKA	0.439	medium relative
CCNB2	0.43	medium relative
RAD51AP1	0.429	medium relative
FOXM1	0.426	medium relative
KIF4A	0.418	medium relative
NUF2	0.412	medium relative
CCNA2	0.402	medium relative
KIF2C	0.401	medium relative
